# Loss of ABC transporters, White, Brown, and Scarlet, prevents increase in mitotic divisions of germline stem cells in response to mating in *Drosophila melanogaster*

**DOI:** 10.1371/journal.pone.0346783

**Published:** 2026-04-07

**Authors:** Megan Pauline Wright, Alisa Vladimirova, Heath M. Aston, Manashree S. Malpe, Cordula Schulz

**Affiliations:** 1 Department of Cellular Biology, University of Georgia, Athens, Georgia, United States of America; 2 Lake Erie College of Osteopathic Medicine, Erie, Pennsylvania, United States of America; 3 Vanderbilt University Medical Center, Nashville, Tennessee, United States of America; Oxford Brookes University, UNITED KINGDOM OF GREAT BRITAIN AND NORTHERN IRELAND

## Abstract

The replenishment of specialized cells depends on the activity of stem cells. Recent advances in stem cell research have shown that the germline stem cells (GSCs) in *Drosophila melanogaster* can increase their mitotic activity in response to mating. Here, we show that this ability to respond to mating is eliminated if the males are mutant for either of the ABC transporters, White (W), Brown (Bw) or Scarlet (St), which are known for their role in eye pigmentation and amine production. However, reducing the expression of *w* specifically from the germline cells also caused a failure to increase GSC mitotic activity upon mating, suggesting that *w* is required intrinsically in the stem cells. The *w* gene is a common genetic background for genetic experiments and frequently used as a control. Our findings underline the importance of careful experimental design and control choice.

## Introduction

As many specialized cells, such as skin, gut, and sperm cells, are lost due to usage or death, the corresponding tissues replenish these through a process commonly referred to as tissue homeostasis [1,2]. One of the key factors in tissue homeostasis is the ability of long-term precursor cells, the stem cells, to undergo mitotic cell divisions. Their daughter cells then either become new stem cells or short-lived precursor cells that undergo a cascade of regulated proliferation and differentiation steps to replace the lost cells [3–6]. To perform tissue homeostasis effectively, stem cells must adjust their mitotic activity to situations of demand, such as injury or growth phases. Indeed, GSCs in the gonad of *Drosophila melanogaster* can adjust their mitotic index (MI^GSC^) to a variety of factors, including diet and temperature [7,8]. Specifically, male GSCs divide more frequently when the males are repeatedly mated to virgin females compared to their sibling males that are not allowed to mate [9]. How this increased demand, caused by repeated mating, is met by the GSCs is not well understood, even though some of the necessary elements have been elucidated. The increase in MI^GSC^ is dependent on the activity of G-proteins and on seven non-redundant G-protein coupled receptors (GPCRs), including three serotonin (5HT) receptors and an octopamine (OA) receptor [9].

In *Drosophila*, the synthesis of bioamines, 5HT, OA, and dopamine (DA), requires the same precursor molecules as the production of eye pigments. For both pathways, these precursors are transported across membranes by the ATP-dependent ABC transporter, W [10,11]. W can form active transporters by heterodimerization with other ABC transporters, two of which have been studied in *Drosophila*, Scarlet (St) and Brown (Bw) [12–17]. In combination with St, W transports tryptophan, which becomes synthesized into 5HT in the neurons and into the brown pigment, ommochrome, in the eye [18,19]. Together with Bw, W transports guanine, the precursor for tetrahydrobiopterine (BH4). BH4 is a co-factor for enzymes that synthesize several amines, including 5HT, DA, and OA in the neurons, and for the production of red eye pigments, specifically drosopepterines [20,21].

A range of other substrates are also transported by W, including guanosine 3’-5’ cyclic monophosphate (cGMP), kynurenine, riboflavin (the vitamin B2 precursor), xanthine, and kynurerine [18–20,22–25]. W, but not its partners, Bw or St, is associated with cholesterol homeostasis for its role in olfactory learning. While the authors did not suggest a direct role for W in cholesterol transport, they suggested that W may form a heterodimer with one of several other unstudied ABC transporters to control the levels of cholesterol and cholesterol esters [26].

Among other tissues, expression of *w* was detected in pigmented cells, the eyes, the malphigian tubules, the testes sheath, and in non-pigmented glia and neurons of the brain [17,27,28]. The W protein was reported in vesicular fractions of several cell types, suggesting a role in intra-cellular transport. Electron-microscopy experiments revealed a localization of W in the membranes of the pigment granules within pigment cells and retinal cells, suggesting that it transports molecules from the cytoplasm into the pigment granules found in vesicles, and in Cos-1 cells, W was detected on the endosomal compartment [24,29]. A potential role for W in cargo influx or efflux via the plasma membrane has neither been shown, nor refuted.

Not surprisingly, animals mutant for *w* display an array of phenotypes. W mutant animals have abnormal vision, including increased sensitivity to light and abnormal phototaxis, and their retinal and dopaminergic neurons degenerate [30–34]. Along with the vision defects, *w* mutant animals display neurological phenotypes, such as learning and place memory defects and defects in locomotion [25,35–37]. *w* mutant flies also have altered sensitivity to anesthetic treatments and alcohol, and are less aggressive than wild-type (wt) flies [38–40].

Here, we show that males homozygous mutant for either of the two commonly used *w* alleles successfully mated but had no significant difference in their MI^GSC^ when compared to their non-mated siblings. In an attempt to determine how the binding partners of W factor into this effect, we found that males homozygous mutant for *bw*, *st*, or double mutant for both also had a similar MI^GSC^ as their non-mated siblings. Hence, it appears that multiple cargos passing the membranes with assistance of the ABC transporters are essential for the increase in MI^GSC^ in response to mating. Reducing *w* specifically from the germline cells resulted in the same failure to increase MI^GSC^, suggesting that cell metabolic processes limit the function of GSCs to respond to a demand for more sperm.

## Results

### Animals mutant for *w* did not increase their MI^GSC^ in response to mating

**Fig 1:**
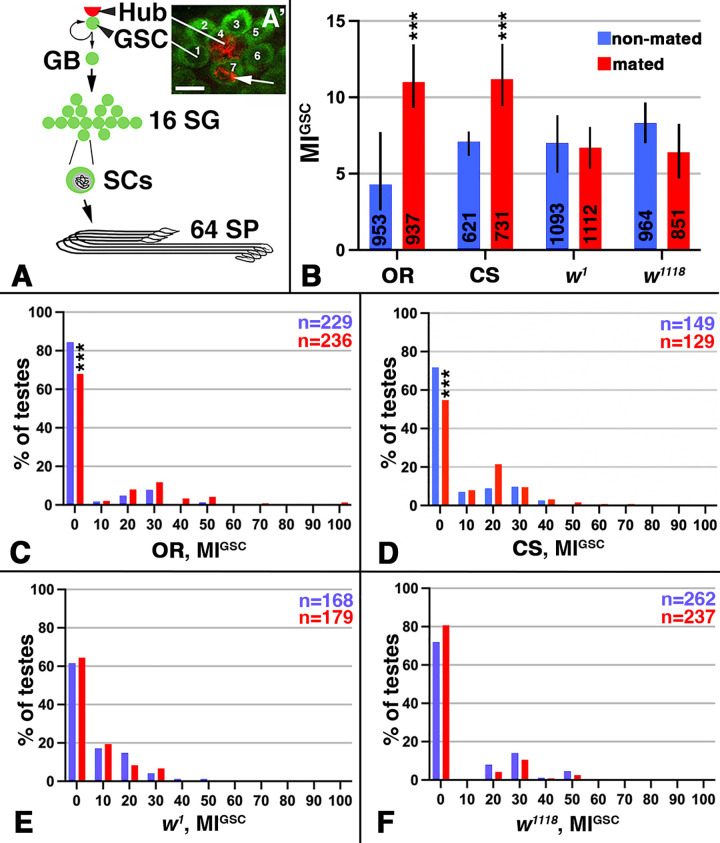
Mated *w* mutant males failed to increase MI^GSC^. A) Cartoon depicting how a GSC division results in a new GSC and a stem cell daughter that will ultimately produce 64 spermatids (SPs). GB: gonialblast, SG: spermatogonia, SC: spermatocyte. A’) Immuno-staining of the apical tip of a wt testis, showing seven Vasa-positive GSCs (green) next to the FasIII-positive hub (red). One of the seven GSCs is in mitosis based on anti-pHH3-staining (red; arrow). Scale bar: 10μm. B-F) Blue: non-mated condition, red: mated condition, ***: P-value < 0.001, numbers of GSCs in bar graphs and number of gonads (n=) in FDGs as indicated. B) Bar graph showing MI^GSC^ of wt and *w* mutant males, as indicated. C-F) FDGs showing median of bin of MI^GSC^ across populations of males on the X-axes and the percentage of testes with each MI^GSC^ on the Y-axes; genotypes as indicated.

In *Drosophila* males, GSCs are found at the tip of the testes where they attach to somatic hub cells (Fig 1A, 1A’). When one of these GSCs divide, the two daughter cells normally take on different fates. One of them becomes a new GSC that remains attached to the hub and replenishes the stem cell pool. The other becomes a gonialblast (GB) that proliferates into 16 spermatogonia. After meiosis, the cells ultimately differentiate into exactly 64 spermatids (Fig 1A) [41,42]. If a demand for more spermatids is produced via mating experiments, the GSCs divide more frequently, which can be measured by investigating the MI^GSC^ using simple immuno-fluorescence experiments [8]. The hub cells can be labeled with an antibody against FasciclinIII (FasIII) and the attached GSCs can be visualized with an antibody against Vasa. The Vasa-positive GSCs are imaged (Fig 1A’) and counted in all focal planes around the hub. By adding an antibody against phosphorylated Histone-H3 (pHH3), cells in mitosis become apparent and the percentage of the GCSs that are actively dividing, the MI^GSC^, can be calculated [8].

While wt males always significantly increase MI^GSC^ in response to repeated mating, we noted considerable inconsistencies in our data when males were kept in a *w* mutant background. Intrigued by this observation, we set out to investigate if the failure of males to increase MI^GSC^ might be due to the *w* mutation itself. We employed two of the commonly used *w* alleles, *w*^*1118*^ and *w*^*1*^, and set up single male mating experiments with these, as well as with two wt strains, Oregon R (OR) and Canton S (CS). To obtain reproducible data, we used 100 males for the mated group and another 100 males for the non-mated controls for each experiment. To illustrate the overall MI^GSC^, we added the GSC numbers and calculated MI^GSC^ from replicate experiments and displayed the differences between non-mated and mated animals in bar graphs. These show that OR and CS males clearly increased MI^GSC^ when repeatedly mated, and that animals mutant for *w*^*1118*^ or *w*^*1*^ displayed similar MI^GSC^ under both conditions (Fig 1B).

A GSC division is not a frequent event. Most animals within one group, or population, have zero GSCs in division. To assure that the differences in MI^GSC^ between non-mated and mated males are not due to a few unusually behaving males, but by an overall MI^GSC^ increase within the group of males, we use Frequency Distribution Graphs (FDGs). FDGs group the males based on different MI^GSC^ and then display how often those MI^GSC^ are represented among the testes of one group of males. In the FDGs, the wt mated males had a significant decrease in the number of GSCs with a MI^GSC^ of zero. Overall, it appears that there were more testes with MI^GSC^ above zero (Fig 1C, 1D). For *w* mutant males, in contrast, we did not observe a reduction in the number of GSCs with a MI^GSC^ of zero and did not see more testes with MI^GSC^ above zero upon mating (Fig 1E, 1F). All groups of males used in our experiments mated with virgin females based on visual observations and the production of progeny (Table 1). We conclude that *w* is required for the increase in MI^GSC^ in response to mating.

**Table 1:** Copulation Success Assay. Male copulation success was calculated based on the % of singly housed females that produced offspring after mating with males of the indicated genotype on day one of the experiment. Note that *bw*^*1*^*st*^*1*^ x *bw*^*1*^*st*^*1*^ males appeared to have mated slightly less. BL#: Bloomington stock number.

Male Genotype	BL #	% of Females that Produced Offspring
OR	N/A	72%
CS	N/A	62%
*w* ^*1118*^	N/A	81%
*w* ^*1*^	679	70%
*bw*^*1*^/*bw*^*2b*^	245/248	79%
*mw*-1	N/A	69%
*mw*-2	N/A	74%
NG4−1	N/A	85%
NG4−2	4937	87%
*bw*^*1*^/*bw*^*1*^*st*^*1*^	245/686	64%
*bw*^*2b*^/*bw*^*1*^*st*^*1*^	248/686	70%
*st* ^*1*^ */bw* ^*1*^ *st* ^*1*^	605/686	77%
*bw* ^*1*^ *st* ^*1*^ */bw* ^*1*^ *st* ^*1*^	686	50%
*w*^*33641*^/NG4–1	33641	76%
*w*^*33644*^/NG4–1	33644	86%

### The *bw* and *st* genes were also required for the increase in MI^GSC^ in mated males

**Fig 2:**
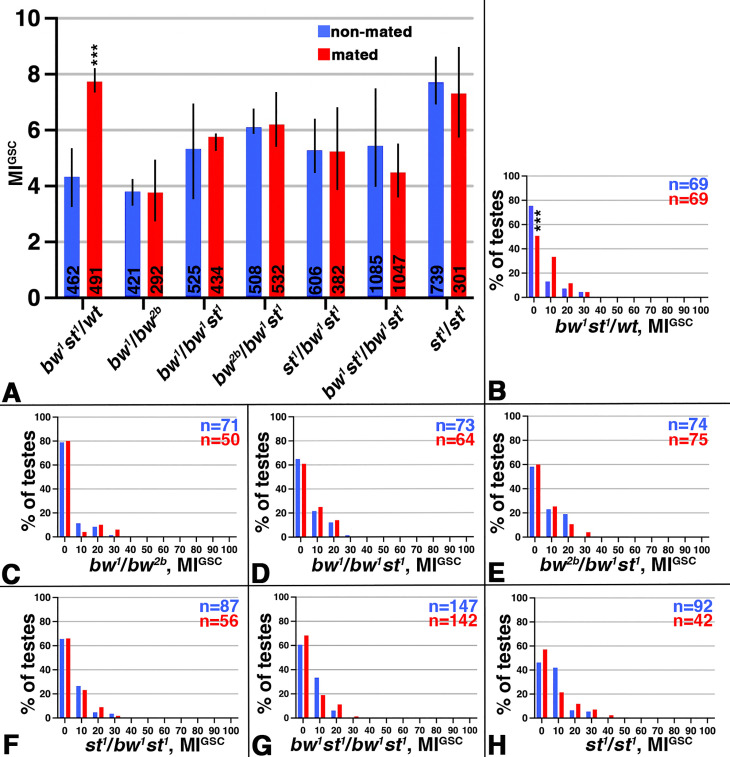
Mated *bw* and *st* males failed to increase MI^GSC^. A-H) Blue: non-mated condition, red: mated condition, ***: P-value < 0.001, numbers of GSCs in bar graphs and number of gonads (n=) in FDGs as indicated. A) Bar graph showing MI^GSC^ of control (*bw*^1^*st*^1^/wt), *bw*, and *st* mutants and double mutants, as indicated. B-H) FDGs showing median of bin of MI^GSC^ across populations of males on the X-axes and the percentage of testes with each MI^GSC^ on the Y-axes; genotypes as indicated.

Since Bw and St are both W’s binding partners, we hoped to pinpoint the activity of W in the response to mating to a specific pathway. Hence, we conducted our mating experiments with *bw* and *st* single, and double mutant males, as listed in Fig 2. As obvious from the bar graph, control males (*bw*^*1*^*st*^*1*^*/*wt) showed the expected increase in MI^GSC^, while all the mutants failed to respond to mating (Fig 2A). In contrast, compared to the control (Fig 2B), the FDGs of the mutants showed no significant decrease in the number of testes at an MI^GSC^ of zero and we did not see more testes with an MI^GSC^ above zero (Figs 2C-H). We conclude that transport via the Bw and St transporters is also essential for the normal GSC behavior in mated males.

### The *mini-white* (*mw*) gene did not restore the ability of *w* males to respond to mating

*mw* is a version of *w* that lacks the first intron and is commonly used as a reporter to demonstrate the presence of a mobile element in the fly genome [27]. Expression of m*w* can restore many defects seen in *w* mutants. For example, expression of *mw* rescued the eye pigmentation and retinal degeneration defects in *w* animals [25]. However, the expression levels of *w* and *mw* underlies position-effect variegation and the insertion in different locations of the genome produce flies with varying eye colors, that range from light yellow to orange to red [43–45]. The *mw* reporter also contains an insulator that can severely reduce its expression levels [46]. To overcome the limitation of expression levels, more than one copy of *mw* is often used for rescue experiments. For example, four copies of the *mw* gene were used to rescue the defects of *w* mutant males in copulation success [47].

**Fig 3:**
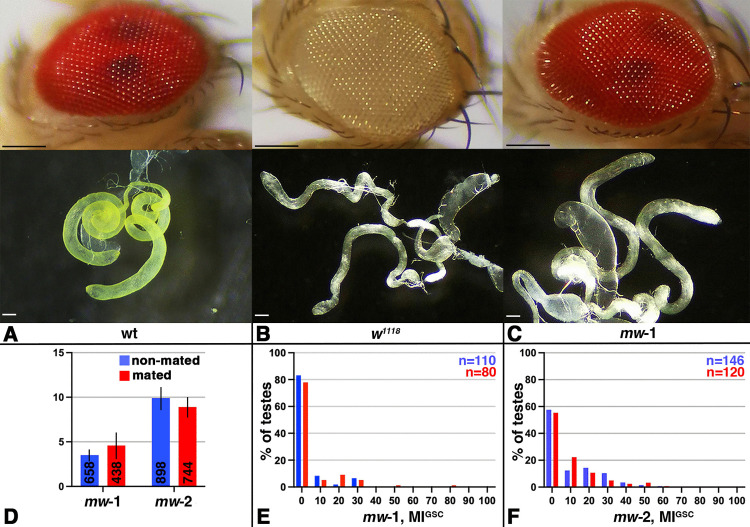
Mated *mw* flies did not bring back the increase in MI^GSC^ upon mating. A-C) Images showing eye color (top) and testes color (bottom) of control and experimental fly lines, as indicated. black scale bar = 250µm and white scale bar = 100µm. D-F) Blue: non-mated condition, red: mated condition, ***: P-value < 0.001, numbers of GSCs in bar graphs and number of gonads (n=) in FDGs as indicated. D) Bar graph showing MI^GSC^ of experimental flies. Compare to wt controls in Fig 1B. E-F) FDGs showing median of bin of MI^GSC^ across populations of males on the X-axes and the percentage of testes with each MI^GSC^ on the Y-axes; genotypes as indicated.

We attempted to rescue the effect that mutations in *w* have on MI^GSC^. For this, we used two fly lines that carry four copies of the *mw* gene (*mw*-1 and *mw*-2, Table 2). These are the same fly lines we use for our mating experiments below, but in a *w* mutant background. As expected, expression of *mw* in these lines restored eye color to a dark red (Fig 3A-C). However, it did not restore testis sheath pigmentation defects (Fig 3A-C), probably because the *mw*-constructs lacks the necessary regulatory elements [27]. When we performed single male mating experiments with either *mw* line, we also did not see a rescue in the response to mating of the *w* mutant males (Fig 3D). The FDG created from this mating trial showed no significant decrease in the number of testes at an MI^GSC^ of zero and no increase in testes with an MI^GSC^ above zero (Fig 3E, 3F). Since we did not succeed in restoring the defects in *w* mutants via *mw* expression, we performed the following experiments with flies that had been crossed into a wt X-chromosomal background (referred to as NG4 flies; Table 2).

**Table 2:** Transgenic flies used in this study. Abbreviation and complete genotypes of fly stocks are shown.

Abbreviation	Genoytype
*mw*-1	w-/Y; UAS-*dicer* (*mw+*)/UAS-*dicer* (*mw+*); *nanos*-Gal4-VP16 (*mw+*)/*nanos*-gal4-VP16 (*mw+*)
*mw*-2	w-/Y; UAS-*dicer* (*mw+*)/UAS-*dicer* (*mw+*); *nanos*-Gal4 (*mw+*)/*nanos*-gal4 (*mw+)*
NG4−1	X/Y; UAS-*dicer* (*mw+*)/UAS-*dicer* (*mw+*); *nanos*-Gal4-VP16 (*mw+*)/*nanos*-gal4-VP16 (*mw+*)
NG4−2	X/Y; UAS-*dicer* (*mw+*)/UAS-*dicer* (*mw+*); *nanos*-Gal4 (*mw+*)/*nanos*-gal4 (*mw+)*
*w* ^*33641*^	*y, v*^*1*^*;* P{TRiP.HMS00042}attP2/TM3, *Sb*^*1*^
*w* ^*33644*^	*y, v*^*1*^*;* P{TRiP.HMS000425attP2

### Reducing *w* from the germline reproducibly blocked the increase in MI^GSC^ in response to mating

**Fig 4:**
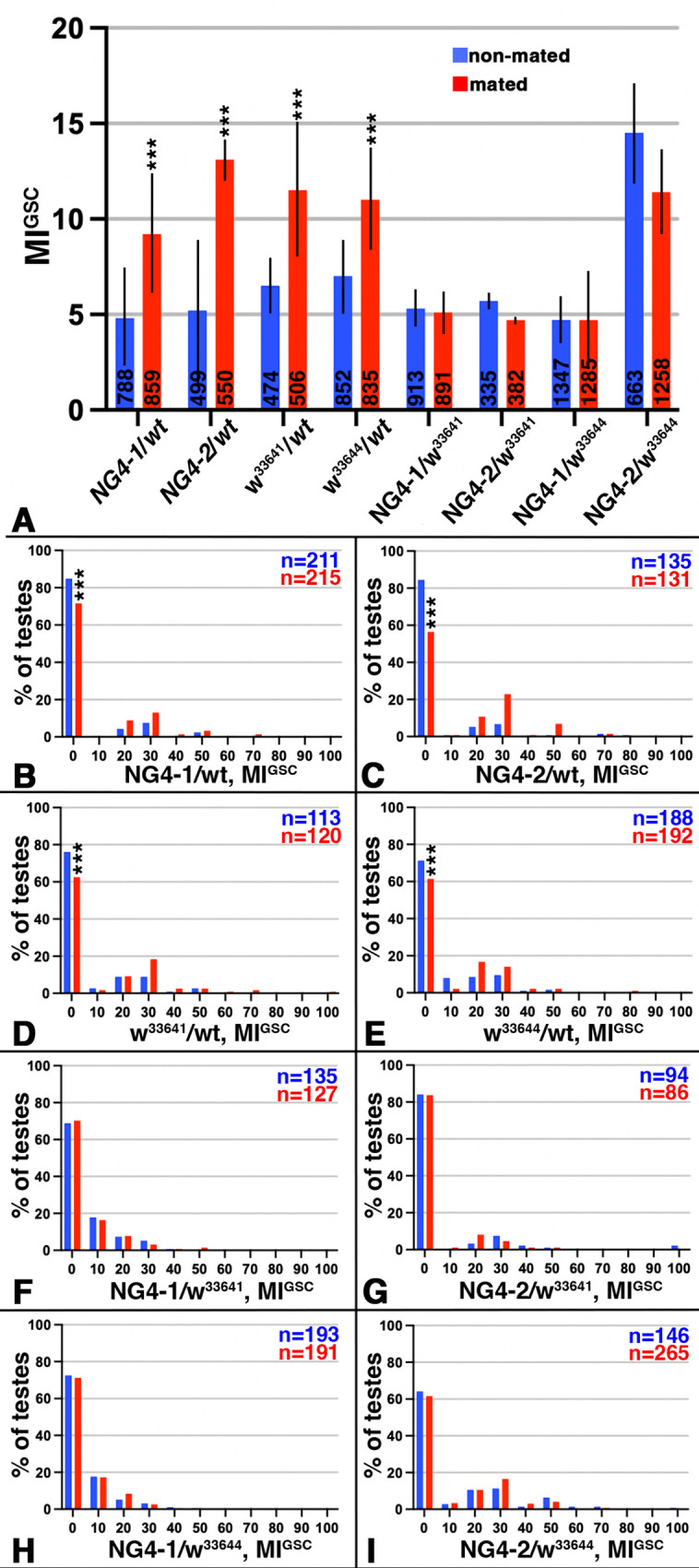
Decreasing the expression of *w* in the germline via RNAi repeatedly lead to a failure to increase MI^GSC^ after mating. A-I) Blue: non-mated condition, red: mated condition, ***: P-value < 0.001, numbers of GSCs in bar graphs and number of gonads (n=) in FDGs as indicated. A) Bar graph showing MI^GSC^ of control (wt crossed to either NG4 or RNAi line) and experimental (either NG4 crossed to RNAi lines), as indicated. B-I) FDGs showing median of bin of MI^GSC^ across populations of males on the X-axes and the percentage of testes with each MI^GSC^ on the Y-axes; genotypes as indicated.

To investigate if the W transporter is dispensible within the germline cells for the increase in MI^GSC^ in response to mating, we used tissue-specific knock-down of *w* via two independent RNA-interference (RNAi) lines (*w*^*33641*^ *and w*^*33644*^; Table 2) [48,49]. To achieve reduction specifically within the germline, we crossed the RNAi flies to NG4−1 and NG4−2 (Table 2). NG4 is a commonly used germline-Gal4-transactivator [50]. NG4−1 and NG4−2 have previously been successfully used for mating experiments [9]. Progeny from wt animals crossed to either of these NG4 or to either of the RNAi lines showed the stereotypical increase in MI^GSC^, but when either of the two *w* RNAi lines were crossed to either of the NG4, their progeny failed to increase MI^GSC^ in response to mating (Fig 4A). Consistent with this, the FDG for the control crosses showed a significant decrease in the number of testes from mated males with a MI^GSC^ of zero and an increase in the number of testes with an MI^GSC^ above zero (Fig 4B-E), while the FDGs for the *w* RNAi crosses did not show it (Fig 4F-I).

## Discussion

The *w* mutation is heavily used as a marker in genetic and molecular studies, but the underlying cellular and biochemical functions of *w* are normally not considered as contributing factors to a phenotype. Yet a variety of abnormal behaviors have been associated with the *w* mutation. Here we show that loss of the W transporter from whole males or reduction specifically in the male germline through RNAi blocked the increase in MI^GSC^ after mating. This failure to respond to mating could not be rescued by expressing several copies of the *mw* gene. A reasonable explanation for this observation is that the *mw* constructs lack a gonad-specific enhancer element or that we could not reach high enough expression levels from the *mw* genes used in this study.

GSCs are neither pigmented cells, nor neurons, suggesting a different function for W within the germline. It is possible that W interacts with an unidentified ABC transporter in the germline to specifically regulate mitotic divisions. However, the pleiotropic effect of *w* on the flies and the variety of substances transported by the W protein suggests that the failure of the mutant males to increase their MI^GSC^ could have multiple underlying reasons.

Interestingly, *w* affects mating behavior. Expression of *w* throughout the male flies from a heat-shock driven transgene, or loss of *w* function, caused increased male sexual arousal and male to male courtship behavior, even in the presence of females [29,51–53]. Loss of *w* also drastically slows copulation success of virgin males, but not so much of mating-experienced males [47,53]. Though we cannot exclude the possibility that loss of *w* has an effect on the fly mating behavior in our experiments, we doubt this is directly linked to their failure to increase MI^GSC^. We performed all experiments with single males (which excludes male to male courtship) and the males were non-virgins, arguing against a reduced copulation success. This is supported by the results of our copulation success assay that showed that *w* mutant males copulated successfully with an similarly large number of virgin females as wt males did (Table 1).

The effects of the *w* mutation outside the eye have been associated with a role for *w* in the nervous system, mediated by 5HT and DA. For example, 5HT is essential for place memory and plays a role in phototaxis, and DA was associated with male aggression [34,36,39]. Consistent with a role for neurotransmitters in *w* phenotypes, several previous studies have shown that *w* mutant animals have abnormal levels of amines, including DA and 5HT [28,36,54]. Mutants for the two known binding partners of W, Bw and St, also contained abnormal amine levels [28]. Together with our observation that these mutants also failed to increase MI^GSC^ upon mating, it opens the possibility that the increase in MI^GSC^ in mated wt males is dependent on amines from the nervous system. This appears consistent with our previous findings that amine receptors are required for the increase in MI^GSC^ in response to mating [9].

In addition to a possible role for *w* in the nervous system in regulating MI^GSC^, we show that loss of *w* from the germline cells also impaired the ability of the mated males to respond to mating. During aging, the intestinal stem cells of *Drosophila* increase the expression of *w*, as well as the frequency of their divisions. Interestingly, loss of *w* reduced intestinal cell proliferation due to a role in metabolism that *mw* did not restore [55]. We did not detect a significant difference in the expression of *w* in testes tips from mated compared to non-mated wt males [56]. However, we agree with the idea of a role for *w* in stem cell metabolism. Likely, any mutation, genetic situation, or environmental factor could reduce the activity of the GSCs, and, hence, interfere with their performance, such as decreasing the frequency of mitotic divisions. We also showed that at least seven GPCRs are required for increasing MI^GSC^ in response to mating [9]. It is tempting to speculate that some of the ligands for these receptors may not be instructive, but rather permissive for stem cell activity.

Overall, we found that the increase in MI^GSC^ after mating is dependent on W, Bw, and St, suggesting that they play some role in regulating GSC divisions, even if this role is potentially permissive. These findings highlight the importance of careful planning of mating experiments, which should all be performed in a wt X-chromosomal background.

## Materials and methods

### Fly husbandry

Flies were raised on a cornmeal/agar diet and kept in incubators at 18°C, with a 12 hour light cycle, and 70% humidity. The OR, CS, NG4−1, NG4−2, and *w*^*1118*^ flies were originally obtained from the Bloomington stock center but have been in the laboratory for more than 10 years, while the *w*^*1*^, *bw*, and *st* alleles and the RNAi-flies were obtained more recently (Tables 1 + 2) [57,58]. *X^X*, *y,w,f*/Y/*shi*^*ts*^ flies were provided by Barry Ganetzky.

### Mating experiments

Mating experiments were performed at 29°C. A timeline experiment showed that males of all ages (1–4 weeks) significantly increased their division frequency in response to mating (S1 Fig). We used one to two week old flies and they were fed with yeast paste on apple juice-agar plates in egg lay containers for 24 hours prior to the experiment. Males were placed into one slot of a mating chamber either by themselves (non-mated) or with three virgin females (mated). The chambers were closed by apple juice-agar lids supplemented with yeast paste. On each of the following two days, females were normally discarded, and each mated male was provided with three new virgin females. Apple juice-agar lids supplemented with yeast paste were replaced daily for both conditions. Due to the high volume of virgin females required for mating experiments, we used 1–2 week old females from the stock *X^X*, *y,w,f*/Y/*shi*^*ts*^ [9]. In this stock, only females hatch when animals are raised at 29°C. The same females were then utilized in the copulation success assay.

### Evaluation *of* mating success

In the first round of the mating experiment for each genotype, 100 of the female flies that were housed with the males were placed into individual vials and presence of progeny was noted after seven days. Males have enough seminal fluid to successfully inseminate 2–3 female virgins [59–61] hence we expected an overall mating success of 60–90%.

### Immuno-fluorescence and microscopy

Animals were placed on ice to immobilize them. Gonads were dissected in Tissue Isolation Buffer (TIB) and immediately placed into a 1.5 ml tube with cold TIB buffer. Gonads were then fixed, stained and imaged as previously described [8,62]. The mouse anti-FasciclinIII (FasIII) antibody (1:10) developed by C. Goodman was obtained from the Developmental Studies Hybridoma Bank, created by the NICHD of the NIH and maintained at The University of Iowa, Department of Biology, Iowa City, IA 52242. The goat anti-Vasa antibody (1:50–1:500) was purchased from Santa Cruz Biotechnology Inc. (sc26877). The rabbit anti-Vasa antibody (1:3000) was a gift from Ruth Lehmann. The rabbit anti-phosphorylated Histone H3 (pHH3) antibodies (1:100–1:1000) were purchased from Fisher (PA5–17869), Millipore (06–570), and Santa Cruz Biotechnology Inc. (sc8656-R). Secondary antibodies coupled to Alexa 488, 568, and 647 (1:1000) and Slow Fade Gold/DAPI embedding medium were purchased from Life Technologies. Staining was observed with a Zeiss Axiophot, and images taken with a digital camera and an apotome via the Axiovision Rel. software.

### Graphic presentations and data analysis

Bar graphs and FDGs were generated with GraphPad prism version 10.2.3 and assembled into composite images using Adobe Photoshop. Error bars seen in the bar graphs represent the lowest and highest values of MI^GSC^ seen across the replicates. FDGs show either testes with zero GSCs in division (0 value) or testes with some GSCs in division divided into bins of 10 (0.1–10%, 10.1–20%, etc.). The GraphPad prism default two-tailed student’s t-test was used to analyze statistical relevance.

## Supporting information

S1 Appendix Data used to construct figures. In the file, phh3 indicates the GSCs stained by the histone 3 antibody, which indicates that they are in division.
(XLSX)

S1 Fig Age does not impact the change in MI^GSC^ in response to mating. Bar graph showing MI^GSC^ of flies of different ages. Blue: non-mated condition, red: mated condition, ***: P-value < 0.001, numbers of GSCs in bar graphs as indicated.
(TIF)
